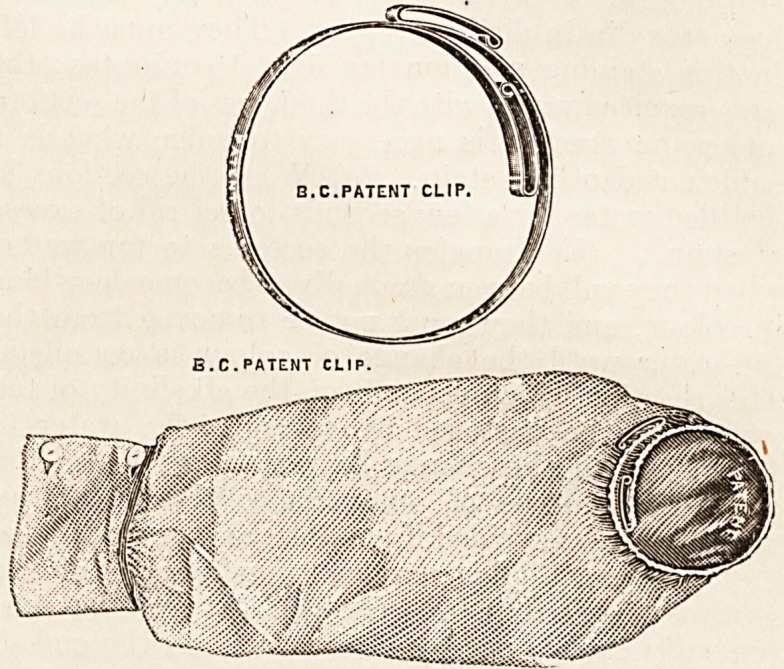# New Appliances and Things Medical

**Published:** 1907-10-12

**Authors:** 


### NEW APPLIANCES AND THINGS MEDICAL.

[We shall be glad to receive at our Office, 28 & 29 Southampton Street, Strand, London, W.C., from the manufacturers, specimens of all new preparations and appliances which may be brought out from time to time.]

### ALLSOPP'S LAGER BEER.

We have received from Messrs. Allsopps, Limited, Burton-on-Trent, specimens of their lager beer, brewed and bottled by themselves. The actual bottles sent to us were so-called half-bottles; but they are fitted with patent stoppers, so that if the whole bottle is not required it will keep fresh and good for several hours after being opened. We are glad to see a firm like Messrs. Allsopp putting on the market in so convenient a form a light, low-fermentation lager beer. We venture to think one of the reasons for what we regard as a regrettable decline in beer-drinking is the backwardness of brewers in moving with the times and producing a light and palatable beer. The so-called lager beers which have been made in this country up till now are thin, acid, and raw in taste, and those standard German beers imported into the country are as a rule freely dosed with preservatives and not infrequently with salicylic acid which often completely spoils their taste. It is a mistake to suppose that lager beer so-called should, because it contains a low amount of alcohol, be thin and watery. Quite the reverse is the case, in that low-fermentation should contain more sugar and dextrine than ordinary English beer. We think Messrs. Allsopp have succeeded in producing a soft, creamy, and thirst-quenching beer of low alcoholic strength, and we feel sure that when the article is thoroughly known it will be appreciated.

### "THERMOS" FLASK.

(Chas. Birchall, Ltd., Victoria Street, Liverpool.)

This flask is a bottle with double walls, from between which the air has been extracted, and by the aid of this apparatus it is possible to keep liquids at the same temperature for about twenty-four hours. Indeed, in the case of cold liquids it is claimed that it has been found easy to prevent any rise in temperature for at least a couple of weeks, though we have not tested this latter statement to this extent. For twenty-four hours, however, we have proved that it is easily possible to keep cold fluids as cold, and hot fluids as hot, as when first put into the bottle, providing only that in the case of hot fluids the bottle is heated before the fluid to be kept is put in it. There must be a great field open for this invention, which cannot fail to prove a comfort to travellers whether well or ill, to doctors on long country drives, to people on picnic, to soldiers on march, and to many others.

### THE "B.C." OVERSLEEVE CLIP.

(Hospitals and General Contracts Company, Ltd., 33 and 35 Mortimer Street, London, W.)

These exceedingly useful clips are made of nickel-plated steel and are therefore easily sterilised. They are of a very simple make, and can be used in hems of oversleeves in place of the elastic or tape ordinarily used. The makers also supply them fixed to a pair of special sleeves, made of a

B.C.PATENT CLIP.

light waterproof material, which can be boiled and perfectly sterilised. We consider the clips a great advance on the contrivances hitherto used for the same purpose; and to the general practitioner, on account of their durability, simplicity, compactness, and lightness, they should prove a boon. The price is 1s. per pair, or, fitted with patent waterproof oversleeve, 3s. 6d. per pair. A slightly larger size is also made for surgeons' use.

### V.D. FRENCH CLARETS, SAUTERNE, AND BURGUNDIES.

(Standring and Drake, 1a New London Street, Mark Lane, E.C.)

We have received samples of this well known brand of French wines. These wines come from the celebrated Verginaud cellars in Bordeaux. Messrs. Standring and Drake, as specimens of the clarets, have sent us bottles of Chateau Talbot, Larose, and St. Julien-Adet. The finest and most expensive of these clarets is the Chateau Talbot. This is a typically good French claret possessing a low alcoholic strength, a low acidity, and a negligible quantity of sugar, and with these chemical properties an exceedingly good flavour and bouquet. The other two clarets are good sound wines, but slightly heavier. The Haut-Sauterne which we have received is undoubtedly a fine wine of full body, containing about 16 vol. per cent. of alcohol and 1½ per cent. of sugar. The Pommard and Volnay which have been sent us are both good types of their class, possessing good soft bouquets and full bodies. We think these wines deserve the reputation they have earned. Their price, varying from 23s. to 36s. per doz. is, we think, a very reasonable one. We can conscientiously advise medical men desiring these types of wines for their patients to recommend the so-called V.D. wines.

## Figures and Tables

**Figure f1:**
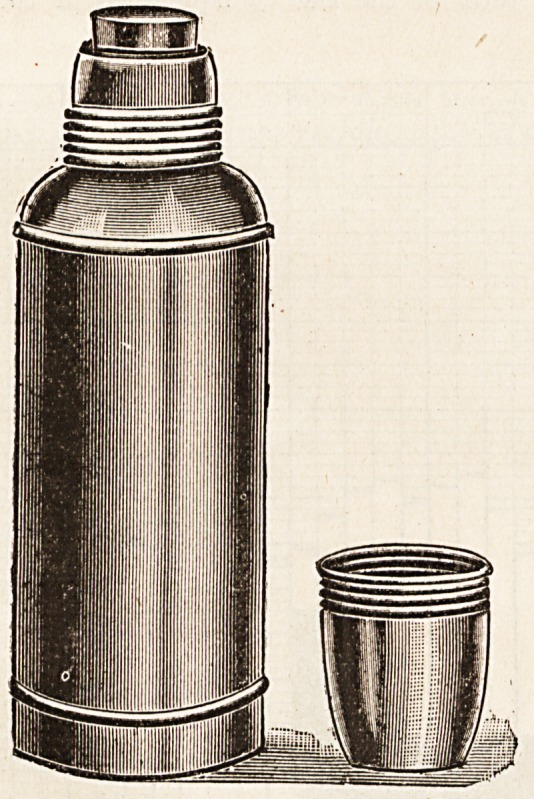


**Figure f2:**